# COVID-19: time for a bold new strategy for medical education

**DOI:** 10.1080/10872981.2020.1764741

**Published:** 2020-05-13

**Authors:** Alastair Watson, Tamsin McKinnon, Scarlet-Daisy Prior, Liam Richards, Christopher A. Green

**Affiliations:** a Birmingham Medical School, University of Birmingham, Birmingham, UK; b Clinical and Experimental Sciences, University of Southampton Faculty of Medicine, Southampton General Hospital, Southampton, UK; c NIHR Southampton Biomedical Research Centre, Southampton Centre for Biomedical Research, Southampton General Hospital, Southampton, UK; d Institute of Microbiology and Infection, University of Birmingham, Birmingham, UK

**Keywords:** Continuing medical education, clinical education, curriculum development/evaluation, professional development, ﻿COVID-19

Dear Editor – COVID-19 has had an unprecedented impact on medical education worldwide leading to the cancellation of lectures, placements, exams, and electives, and ultimately the closure of medical schools[1]. The consequences of this disruption for the development of medical students, their mental health, and preparedness for life as doctors are still to be determined[2]. Moreover, with social distancing in the UK predicted to extend until at least the development of a successful vaccine[3], global changes to medical education are going to be required. This will bring with it new challenges but also unique learning opportunities for medical students and potential for change[4].

Within this crisis, medical students have mobilised on mass to support healthcare systems worldwide[5]. Alongside the UK final year medical students who were graduated early to join the front-line doctors, the University of Birmingham alone has seen over 700 medical students volunteer to support the NHS, something which we have been a part of. Many of us have taken up essential resident clinical roles or have responded to the Chief Medical Officer’s call to deliver urgent public health research studies with the potential to change the epidemic of this disease. This has provided a plethora of unique opportunities for self-directed learning in a range of clinical and research settings. These experiences will undoubtedly benefit the training and development of medical students of all years. Reflecting on these experiences and how they can be incorporated into restructured medical education programmes for the training of our future doctors is now essential[4].

Medical schools have begun to adapt to the pandemic, increasing the provision of distance support, teaching, and examinations using electronic resources and learning environments, which have been increasingly implemented in recent years[6]. However, this will need to be coupled with a rapid risk-assessed remodelling of teaching within a clinical environment in a proactive manner. We call for a fast response from universities and the GMC to reflect on current potential learning opportunities for medical students and to develop a flexible, bold strategy to reshape medical education to rise to the current challenges and new opportunities. Alongside a coordinated mental health and pastoral support plan, this will be key in delivering effective medical education for the development of our future doctors during the ongoing pandemic.
